# Factors Influencing Depressive Symptoms in Middle-Aged South Korean Workers by Job Type: A Population-Based Study

**DOI:** 10.3390/ijerph192114310

**Published:** 2022-11-02

**Authors:** Myoungjin Kwon, Sung Yun Ahn, Sun Ae Kim

**Affiliations:** 1 Department of Nursing, Faculty of Health and Medical Sciences, Daejeon University, Daejeon 34520, Korea; 2 Department of Nursing, Faculty of Life and Health Sciences, Pai Chai University, Daejeon 35345, Korea; 3 Department of Nursing, Faculty of Health and Life Science, Korea National University of Transportation, Chungju-si 27909, Korea

**Keywords:** depressive symptoms, occupations, classification

## Abstract

Depressive symptoms can be influenced by different factors, including job types. In this study, we identified factors that affect depressive symptoms among South Korean workers by job type using data from the Korea National Health and Nutrition Examination Survey. Examined respondents were between 40 and 69 years (N = 9375). A complex sample linear regression analysis revealed various significant factors based on job type. For office workers, the number of household members, health-related quality of life, diabetes, sitting time, subjective health, and stress were significant influencing factors for depressive symptoms (explanatory power of 23.2%) (*p* < 0.001). For service workers, gender, health-related quality of life, food intake, aerobic exercise, sitting time, subjective health, and stress were significant influencing factors for depressive symptoms (explanatory power of 49.6%) (*p* < 0.001). For labor workers, gender, type of living, health-related quality of life, BMI, weight change, weight control, aerobic exercise, diabetes, subjective health, and stress were significant influencing factors for depressive symptoms (explanatory power of 35.8%) (*p* < 0.001). These differences highlight the need for customized programs targeted at each job type to maintain and promote mental health among workers.

## 1. Introduction

A job is an important factor in realizing personal growth [1]. From a positive perspective, a job can enrich an individual’s life by endowing them with importance, power, and control. However, when a job consumes excessive time or creates tension and stress, negative physical and mental aspects can emerge. Job stress is associated with increased depressive symptoms [2]. These conditions have continued to worsen due to successive economic crises, including a global crisis that has spurred various changes in the workplace, entailing heavy competition and an increased demand for proficiency [3,4]. In this context, higher stress can cause physiological, psychological, and behavioral changes, while diminishing health and the overall quality of life [5], all of which may catalyze negative health behavior and depression [6]. Furthermore, previous studies have shown that depressive symptoms and disorders can emerge through increased working days [7], while depression caused by job stress can directly result in lower productivity [8]. Job distress is related to health problems as well as economic costs and is treated as an important issue in occupational health [9]. Further, depression can be a major cause of suicide and is also associated with work-related suicides [10].

Here, it is crucial to note that workers’ physical, psychological, and social health are more important than job efficiency. An excessive emphasis on efficiency may neglect the health of workers who are critical human resources for ensuring productivity. In the case of China, the prevalence of mild to moderate depressive symptoms in the working population was reported as 60.3% [11]. To make matters worse, 71% of those who have depression attempt to hide their illness while at work [12]. Both percentages indicate that depression is a serious issue in the workplace, especially since many workers may not receive adequate treatment because the illness remains undiagnosed.

Although depression is a significant concern for workers of all ages, a previous study revealed that adults were more likely to experience depressive symptoms due to tasks and crises rather than experience issues resulting from internal factors, such as individual personality traits or genetic predispositions [13]. This makes it highly important to examine how job-related factors influence depressive symptoms in this demographic. Individuals over the age of 40 are not only confronted with the need to prepare for old age but must also focus on present issues such as raising children and establishing their independence. Meanwhile, sexual ability and physical strength tend to decrease alongside general functional decline, with a markedly increased potential for developing chronic disease. In sum, middle age is an important psychosocial period in which many individuals experience burdens related to their children, marriage, job changes, future retirement, and the need to take care of aging parents [14]. At this time, a variety of complications increase the likelihood of developing and living with chronic depression [15]. Therefore, life after the age of 40 is significant as it is a period when risk factors for mental health begin in earnest.

As the literature suggests, depression affects life and work in various ways [16]. In most countries, depression is a common chronic disease that reduces the quality of life [17]. Moreover, the prevalence of depression is gradually increasing [18], and workers with depression are 3.05 times more likely to have suicidal thoughts than workers without depression [19]. An extensive body of research also suggests that a large number of personal factors can influence depression, including job stress [20], job satisfaction [21,22], income, physical health, education, marital status, poor living environments [23], smoking, passive smoking [24], alcohol abuse, frequent heavy drinking [25], exercise habits [26], eating behaviors, obesity [27], subjective health status perception, and life satisfaction [28]. Researchers have targeted these issues among specific samples (e.g., medical personnel and certain job groups [29]) or compared influences based on factors, such as employment hours [30] and gender [31]. A previous study on job type tended to focus on details such as motivation [32]. In other words, most of the existing studies have focused on occupational conditions rather than specific job types, such as those in the white-collar (including professionals), service, and labor (including agricultural, forestry, fishery, craftspeople, and simple laborers) industries. To the best of our knowledge, no study has identified and compared factors affecting depressive symptoms according to specific job types. Therefore, our understanding regarding the overall depressive symptoms of current workers is limited. Moreover, in the case of worker suicide, the Center for Disease Control’s (CDC) Preventing Suicide: A Technical Package of Policy, Programs, and Practices [33] suggests strategies for each workplace setting. This is based on a detailed analysis of suicide by occupation type. However, to gain a better understanding, the factors influencing depression, a major factor related to suicide, must first be analyzed by occupation type. Based on the analysis results, interventions and policy alternatives to reduce workers’ depression can be established.

Based on the current literature, we predicted that different factors would influence the depressive symptoms of workers depending on their job type. This demographic is especially important to consider in this context as workers play the most important role in the functioning of their industries. Particularly, the identification of various pertinent factors would be highly useful in the development of interventions aimed at mitigating depressive symptoms for workers in specific job categories. These strategies are expected to provide relief at the individual and group levels, increase organizational productivity, and improve health-related quality of life across the workforce. While many existing studies have targeted a specific city, gender, or occupational group, this study investigates diverse occupations from the Korea National Health and Nutrition Examination Survey (KNHANES), a large population-based study. By using a national health dataset which can be compared across countries, the results have the advantage of being generalizable as the data are representative of all workers in Korea.

This study sought to identify the factors that affect depressive symptoms in middle-aged workers based on their job type, thus providing basic data for interventional strategies targeted at depressive symptoms.

## 2. Materials and Methods

### 2.1. Participants

This study is based on a secondary data analysis. The KNHANES collects representative and reliable populational data on health, health behaviors, and food and nutritional intake at both the national and provincial levels. It is a nationwide health and nutrition survey that aims to accumulate basic data for use in the creation of health policies and goal setting/evaluation for the National Health Promotion Comprehensive Plan and health promotion program development. The planning and execution of the survey were conducted by the Ministry of Health and Welfare and the Korea Centers for Disease Control and Prevention (KCDC).

The KNHANES is data collected by the KCDC since 1998 using a multi-step clustering probability sampling method to generalize for the entire population.

The data were extracted from the second year of the sixth period (2014) and the first and third years of the seventh period (2016 and 2018) of the KNHANES. There were 23,692 total KNHANES respondents for the period investigated in this study (i.e., 2014, 2016, and 2018). Of these, we focused on the 9375 respondents (aged 40–69 years) who answered the questionnaire item on job type. Respondents were further subdivided as follows: 3981 white-collar workers (office workers, managers, experts, and related workers), 2271 service workers (service and sales workers), and 3123 labor workers (including agricultural, forestry and fishery, craftspeople, and simple laborers).

### 2.2. Study Variables

Study variables were selected by referring to previous studies, and the selected variables were classified into sociodemographic, physical, and psychological characteristics based on literature reviews on the risk factors for depression [34].

### 2.3. Sociodemographic Characteristics

Sociodemographic characteristics included gender, age, household income, education level, type of living, number of household members, housing type, homeownership, health-related quality of life, and food intake. Sex was categorized as male or female. Age was categorized into three groups: 40s (40–49), 50s (50–59), and 60s (60–69). Household income was classified as upper, middle, or lower. Education level was classified as ≤middle school, high school, or college. The type of living was categorized as living with a spouse or other partner. The number of household members was categorized as 1, 2, or ≥3. The housing type was classified as house, apartment, or other. Homeownership was classified as yes or no. Health-related quality of life was measured using the EuroQol-5Dimension (EQ-5D) index, which subjectively evaluates current health status based on the following five items: exercise ability, self-management, daily activities, pain/discomfort, and anxiety/depression. The EQ-5D index unifies each item and is calculated by applying the unique health-related quality of life weights for Koreans, as suggested by the Korea Centers for Disease Control and Prevention [35]. In other words, the EQ-5D index calculates the health status of 243 health conditions across five domains as a single quantitative value, with higher values indicating a higher health-related quality of life [36].

### 2.4. Physical Characteristics

Disease-related characteristics included body mass index (BMI), weight change over one year, weight control over one year, drinking, smoking, aerobic exercise, hypertension, diabetes, dyslipidemia, and sitting time.

The BMI was categorized as less than 18.5 kg/m^2^, 18.5 kg/m^2^ to less than 25 kg/m^2^, and more than 25 kg/m^2^. Weight change over one year was classified as an increase, a decrease, and no change, while weight control over one year was classified as a decreased effort, an increased effort, a maintenance effort, and no effort. Drinking and smoking were classified by whether the respondents were currently engaged in these activities or not. Engagement in aerobic exercise was determined based on moderate-intensity physical activity for 2 h and 30 min or more, high-intensity physical activity for 1 h and 15 min or more, or a mixture of moderate-intensity and high-intensity physical activity (1 min of high-intensity is 2 min of moderate-intensity). The presence/absence of hypertension, diabetes, and dyslipidemia was determined based on respective diagnoses from doctors. Sitting time was considered the total amount over the course of a day.

### 2.5. Psychological Characteristics

Psychological characteristics included subjective health, subjective body image, stress, and depressive symptoms. Subjective health was classified as healthy, moderate, or unhealthy, while subjective body image was classified as thin, moderate, or obese. Depressive symptoms were measured using the Patient Health Questionnaire-9 (PHQ-9) developed by Spitzer et al. [37]. It was developed to evaluate mental health in primary healthcare centers using nine items that are answered on a 4-point Likert scale (0 = not at all, 3 = strongly agree). Total values may thus range from 0 to 27 points, with higher scores indicating more severe depressive symptoms. General life stress was categorized as feeling less and feeling much.

### 2.6. Ethical Considerations

The KNHANES periods investigated in this study were approved by the Research Ethics Review Committee of the Korea Centers for Disease Control and Prevention. In compliance with the Personal Information Protection Act and Statistics Act, the Korea Centers for Disease Control and Prevention only provides de-identified data to prevent the identification of individual respondents based on their survey data. Further, only limited data usage is permitted and solely for academic research purposes. Accordingly, this study obtained an ID and password from the Korea Centers for Disease Control and Prevention to download the relevant data. This also provided access to the “Guidelines for the use of the National Health and Nutrition Examination Survey”.

### 2.7. Analysis

This study’s analysis reflected the design of the complex sample recommended by the Korea Centers for Disease Control and Prevention Guidelines. To reduce the hidden bias and increase the sensitivity of the study, sampling weights were assigned according to the guideline of the Korea Centers for Disease Control and Prevention and the suggested analysis procedure was followed. Missing data were statistically excluded. This study identified the factors influencing depressive symptoms based on data extracted from respondents in their 40s, 50s, and 60s who answered the questionnaire item on job type during the investigated KNHANES study periods (see Section 2.1). Using the IBM SPSS 25.0 program (IBM Corp, Armonk, NY, USA), this study assigned and analyzed variable weights presented by the Korea Centers for Disease Control and Prevention, with significance determined at 0.05. In this study, complex sample analyses were performed by assigning weights (the primary sampling unit (PSU), stratification (kstrata), and weight variables) as recommended by the Korea Centers for Disease Control and Prevention.

Sociodemographic characteristics and the degrees of physical and psychological factors were analyzed using frequencies and percentages. While actual values were used for frequencies, numerical values considering weights were used for percentages. Differences in sociodemographic and physical/psychological characteristics were analyzed according to job type using the complex sample x^2^-test and ANOVA, while factors affecting depressive symptoms were analyzed using the complex sample linear regression analysis.

## 3. Results

### 3.1. Sociodemographic Characteristics by Group

There were significant differences based on job type for all demographic and social characteristics (*p* < 0.05). There were more women in service positions, but more men in labor positions. Middle-aged respondents in their 40s were most prominent in office work, while respondents in their 60s were most prominent in labor jobs. Office workers had the highest income levels, while labor workers had the lowest education levels. The rates of both living with a spouse and the number of household members were the highest for office workers. Most office workers lived in apartments but also reported the highest rate of house ownership. Office workers reported the highest overall health-related quality of life. Finally, white-collar workers reported the highest amount of food intake per day, while service workers reported the lowest (Table 1).

### 3.2. Physical and Psychological Characteristics

There were significant differences based on job type for all physical and psychological characteristics (*p* < 0.001). For physical characteristics, the ratio of ≥25 kg/m^2^ BMI was highest among labor workers, but both one-year weight gain and effort to control weight gain were highest among office workers. Drinking was also highest among office workers, but smoking was highest among service workers. Aerobic exercise was most common among office workers but least common among labor workers, who had the highest rates of hypertension, diabetes, and dyslipidemia. However, sitting time was highest among office workers. For psychological characteristics, white-collar workers were more likely to perceive themselves as healthy, while labor workers were the least likely. As for subjective body image, service workers had the highest percentage of obesity, while labor workers had the highest percentage of thinness. Finally, stress was more frequent among labor workers, while depressive symptoms were more frequent among service workers (Table 2).

### 3.3. Factors Affecting Depressive Symptoms Based on Job Type

A complex sample linear regression analysis was performed to identify the characteristics that affect depressive symptoms according to job type. The sociodemographic and physical/psychological characteristics that differed according to job type were set as the independent variables, while depressive symptoms were set as the dependent variable (Table 3).

For office workers, the number of household members, health-related quality of life, diabetes, sitting time, subjective health, and stress were significantly associated with depressive symptoms. Depressive symptoms increased in cases of two household members when compared to three or more but decreased as health-related quality of life increased. They were lower in participants without diabetes than in those suffering from it. Longer sitting times were associated with higher depressive symptoms, which decreased with higher subjectively perceived health and less stress.

For service workers, gender, health-related quality of life, food intake, aerobic exercise, sitting time, subjective health, and stress were significantly associated with depressive symptoms. Male respondents had lower rates of depressive symptoms than female respondents. Depressive symptoms decreased as health-related quality of life and food intake increased. Meanwhile, respondents who did not perform aerobic exercise had lower rates of depressive symptoms than those who did. Depressive symptoms increased as sitting time increased but decreased with higher subjective perceptions of health. Finally, depressive symptoms were lower among those with less stress.

For labor workers, gender, type of living, health-related quality of life, BMI, weight change, weight control, aerobic exercise, diabetes, subjective health, and stress were significantly associated with depressive symptoms. Male respondents reported lower rates of depressive symptoms than female respondents. Those who lived with spouses had lower depressive symptoms than those who did not. Depressive symptoms decreased as health-related quality of life increased but increased as BMI decreased. Participants who lost weight over one year had higher rates of depressive symptoms than those who gained weight or had no change. Meanwhile, those who tried to gain weight over one year had higher rates of depressive symptoms than those who did not try to control their weight. Participants who did not perform aerobic exercise had lower rates of depressive symptoms than those who did. Those without diabetes had lower rates of depressive symptoms than those with the condition. Finally, depressive symptoms decreased with higher subjectively perceived health and less stress.

## 4. Discussion

This study aims to identify factors affecting depressive symptoms according to occupational characteristics using data from the KNHANES with a target of middle-aged participants (40–69 years). The study findings suggest that service workers had higher depressive symptoms than office and labor workers, and it was confirmed that different variables influenced depressive symptoms according to occupational characteristics.

The result of this study reveals an increase in depression due to emotional labor due to the occurrence of surface acting. This can be explained by a previous finding that the depression level of the interpersonal service workers group was high [38]. Since depression was a partial mediator between surface acting and job stress [38], high depression among service workers could be attributed to many surface acting. This can also be explained by a previous finding that women who hide their emotions have a higher risk of depression than those who do not [39].

In this study, health-related quality of life, subjective health, and stress had a common effect on depressive symptoms in office workers, service workers, and labor workers, and each factor had different effects on depression depending on job type. That is, service workers reported the lowest depressive symptoms as their health-related quality of life increased, and the less stress they received, the most depressive symptoms decreased the most. Depression symptoms decreased the most as the office workers perceived that they were subjectively healthy.

As measured via the EQ-5D, this study’s findings on the relationship between health-related quality of life and depressive symptoms appeared in the same context as previous findings in Ethiopia [40] in which patients with an early onset of depression and a longer follow-up duration (greater than 5 years) had a markedly diminished perception of their health-related quality of life. The results of this study can be explained by a previous finding that chronically high depressive symptoms increase the probability of being dissatisfied with health-related quality of life [40].

Additionally, the results of this study indicating that higher stress levels increase depressive symptoms are consistent with the results of previous studies [41] on job stress significantly impacting the development of depressive symptoms. This can be explained by a previous finding that acutely stressful tasks increase cardiovascular reactions such as heart rate variability and peripheral blood flow, which especially increase the risk of mental illness [42].

Generally, a poor health status is typically associated with increased levels of depression. Consistent with previous research [43], this study found that poor subjective health was associated with higher rates of depressive symptoms. Service workers in this study reported the lowest symptoms of depression as their health-related quality of life increased. The characteristics of service workers with low income and a lack of regular workers could explain this finding. Referring to the results of a previous study [44] health-related quality of life is high when the income is high and regular workers are high. Additionally, since the service jobs received less stress than the other occupations, their depression symptoms decreased the most, and stress is highly correlated with health-related quality of life [45].

The finding that depressive symptoms decreased the most as the office workers perceived themselves as being subjectively healthy was partially consistent with a previous study targeting only married women working in offices which found that subjective health status affects mental health variables [46]. However, this was not compared with other occupations; therefore, more research is needed.

Although there is a difference in the degree for each type of occupation, results indicate that health-related quality of life, subjective health, and stress appear to have a common impact on depressive symptoms among middle-aged workers across all job types. This highlights the need to target some combination of all such aspects when attempting to reduce depressive symptoms stemming from the workplace. This study also found that sitting time affected depression for office and service workers, which can be explained by the fact that long sitting time as an occupational characteristic affects depressive symptoms in office and service workers [47]. Labor workers perform tasks that are largely based on physical activity and they spend less time sitting, therefore their tasks are relatively less likely to affect depressive symptoms.

On the other hand, a previous study [48] revealed that the simple labor group, which comprises jobs with a lot of physical activity, was more likely to belong to the passive attitude group. Therefore, it is difficult to interpret the short amount of time sitting labor workers as simply desirable. Occupational activities alone account for the majority of their physical activity, which may imply a lack of leisure physical activity compared to other occupations. Therefore, more research is needed on the sitting time of labor workers. Although other job types may require extensive sitting time due to the nature of the work, employees should be given opportunities to reduce these amounts to maintain proper mental health.

Diabetes was also found to affect depressive symptoms among office and labor workers. Notably, diabetes mellitus is a risk factor for the onset of depression because it causes numerous complications in the disease process. Findings that diabetes affects depression are supported by the results of previous studies in Nepal reporting that severe depression in type 2 diabetic patients decreased their social relationship domain score [49]. However, the condition was not a statistically significant factor for depression among service workers. The reason why it was not statistically significant for service workers might be due to the characteristics of service jobs with many interpersonal and social relationships. This highlights the need to address the potential lack of social relationships among office and labor workers. Since this study is a secondary data analysis study, it is necessary to study interpersonal relationships and depression in diabetic patients to determine the detailed cause.

Gender and aerobic exercise were found to affect depressive symptoms among service and labor workers. As service jobs may entail emotional factors and labor jobs often require increased physical strength, gender cultures and related characteristics may have greater impacts on job contents when compared to the demands of office jobs. In this study, males in service and labor jobs had lower rates of depressive symptoms. The findings of this study can be explained by a previous finding that men tend to have increased levels of stress and anxiety according to the contents and/or quantities of their work, whereas women tend to experience higher levels of interpersonal stress and depression according to the character of their typical working conditions [50].

Aerobic exercise was found to affect depressive symptoms among service and labor workers. Many previous studies have reported that aerobic exercise reduces depression [51,52]; however, this study atypically found that service and labor workers who did not perform aerobic exercise had lower rates of depressive symptoms than those who did. This may be because service and labor jobs already entail the need for extensive physical activity. In this case, excessive aerobic exercise may be problematic. However, additional research is needed to confirm this issue.

Among labor workers, depressive symptoms were also associated with decreased BMI, losing weight over the previous one-year period, and attempts to gain weight. These issues may be due to concomitant decreases in physical strength, which is more essential in labor work due to the unique job demands. Meanwhile, labor workers were also the most likely to perceive themselves as unhealthy and/or skinny. This emphasizes the need to focus on physical strength and appropriate weight maintenance when attempting to manage depressive symptoms among workers in the labor industry.

Despite its strength, this study has a limitation. While the data used in this study were highly representative and reliable due to the large-scale nature of the KNHANES, this investigation was limited in that its limited statistical analyses were based on secondary statistical processing. This points to the need for additional research involving variables that were not already included in the dataset and various statistical methods.

## 5. Conclusions

This study, using the KNHANES data with a target of middle-aged participants (40–69 years), was conducted under the assumption that the factors affecting depressive symptoms would be different according to each occupational characteristic, such as office jobs, including professional jobs; service jobs, which require a lot of interpersonal interactions; and labor jobs, which emphasize physical activity. As a result, it is meaningful to identify variables that have different effects on depressive symptoms according to occupational characteristics of middle-aged people. Since these variables significantly varied according to job type, the results can be used as basic data in the development of tailored programs that more accurately target mental health issues for workers in different industries. However, since the data used in this study were extracted from data representing the South Korean population, there is a limitation in generalizing it to other cultural or national contexts. The results of this study can be generalized only in South Korea. Through this study, future studies should investigate the effects of variables that depend on certain job characteristics rather than focusing on jobs as a single concept.

## Figures and Tables

**Table 1:** Participant sociodemographic characteristics (N = 9375).

Characteristics		Office Work N (Weight %)/M (SE)	Service Work N (Weight %)/M (SE)	Labor N (Weight %)/M (SE)	x^2^/F (*p*)
Gender	Male	2046 (58.1)	779 (41.9)	1944 (69.4)	401.73 (<0.001)
	Female	1935 (41.9)	1492 (58.1)	1179 (30.6)	
Age (years)	40–49	1167 (59.3)	517 (40.6)	577 (32.1)	397.44 (<0.001)
	50–59	653 (32.5)	630 (43.8)	865 (44.0)	
	60–69	241 (8.2)	304 (15.6)	722 (23.9)	
Household income	Upper	1552 (37.4)	473 (20.0)	488 (15.2)	569.73 (<0.001)
	Middle	1887 (47.9)	1224 (53.4)	1802 (57.4)	
	Lower	537 (14.7)	567 (26.6)	830 (27.4)	
Education level	≤Middle school	88 (1.8)	591 (21.3)	1367 (35.4)	3380.12 (<0.001)
	High school	862 (21.8)	1098 (51.0)	1261 (46.0)	
	≥College	3030 (76.4)	581 (27.7)	494 (18.6)	
Type of living	Living with spouse	2884 (95.9)	1521 (86.6)	2283 (85.2)	235.09 (<0.001)
	Others	141 (4.1)	267 (13.4)	490 (14.8)	
Number of household members	1	285 (7.8)	182 (6.9)	409 (10.8)	134.51 (<0.001)
	2	731 (16.7)	580 (22.0)	944 (25.8)	
	≥3	2965 (75.6)	1509 (71.0)	1770 (63.4)	
Housing type	House	836 (23.0)	779 (34.5)	1121 (36.4)	322.85 (<0.001)
	Apartment	2742 (66.1)	1165 (50.3)	1494 (46.2)	
	Others	403 (10.8)	325 (15.2)	508 (17.4)	
Homeownership	No	1200 (32.2)	715 (32.5)	1050 (35.7)	10.42 (0.018)
	Yes	2781 (67.8)	1553 (67.5)	2070 (64.3)	
EQ-5D		0.98 (0.001)	0.96 (0.002)	0.96 (0.001)	106.84 (<0.001)
Food intake (g)		1824.95 (14.64)	1587.84 (18.07)	1679.36 (19.90)	55.48 (<0.001)

EQ-5D: EuroQol-5Dimension.

**Table 2:** Participant physical and psychological characteristics (N = 9375).

Characteristics		Office Work N (Weight %)/M (SE)	Service Work N (Weight %)/M (SE)	Labor N (Weight %)/M (SE)	x^2^/F (*p*)
Body Mass Index (kg/m^2^)	<18.5	174 (4.6)	84 (4.9)	64 (2.6)	49.19 (<0.001)
	18.5–24.9	2197 (63.0)	1186 (62.7)	1533 (58.0)	
	≥25	1070 (32.4)	625 (32.5)	1060 (39.3)	
Weight change	Gain	1078 (27.6)	575 (26.8)	537 (19.4)	81.46 (<0.001)
	Loss	467 (12.6)	310 (15.0)	391 (12.9)	
	No change	2415 (59.8)	1316 (58.2)	2148 (67.7)	
Weight control	Decrease	1888 (47.0)	1035 (45.7)	1100 (36.4)	139.64 (<0.001)
	Increase	155 (4.1)	95 (4.7)	213 (7.0)	
	Maintain	787 (19.0)	433 (18.9)	528 (16.9)	
	No effort	1145 (29.9)	698 (30.8)	1266 (39.7)	
Drinking	No	1374 (31.8)	865 (34.7)	1261 (36.2)	15.56 (<0.001)
	Yes	2601 (68.2)	1396 (65.3)	1845 (63.8)	
Smoking	No	876 (49.0)	323 (37.4)	817 (45.1)	30.74 (<0.001)
	Yes	830 (51.0)	486 (62.6)	880 (54.9)	
Aerobic exercise	No	1929 (46.3)	1171 (49.7)	1711 (53.0)	30.98 (<0.001)
	Yes	2046 (53.7)	1081 (50.3)	1391 (47.0)	
Hypertension	No	3592 (91.4)	1804 (84.9)	2304 (79.4)	209.31 (<0.001)
	Yes	374 (8.6)	407 (15.1)	791 (20.6)	
Diabetes	No	3842 (96.8)	2125 (94.5)	2820 (92.3)	70.57 (<0.001)
	Yes	139 (3.2)	146 (5.5)	302 (7.7)	
Dyslipidemia	No	3617 (91.9)	1894 (88.1)	2517 (83.9)	109.58 (<0.001)
	Yes	349 (8.1)	317 (11.9)	578 (16.1)	
Sitting time (h/day)		9.15 (0.070)	7.45 (0.189)	7.67 (0.150)	71.98 (<0.001)
Subjective health	Healthy	1488 (37.2)	684 (31.2)	836 (28.8)	88.57 (<0.001)
	Moderate	2080 (52.0)	1231 (53.5)	1708 (54.9)	
	Unhealthy	413 (10.8)	356 (15.3)	579 (16.3)	
Subjective body image	Thin	563 (14.4)	281 (14.0)	522 (16.5)	19.42 (<0.001)
	Moderate	1571 (39.1)	883 (37.8)	1266 (40.8)	
	Obese	1841 (46.5)	1097 (48.2)	1319 (42.7)	
Stress	Feel less	2716 (68.0)	1585 (70.2)	2409 (76.9)	69.95 (<0.001)
	Feel much	1259 (32.0)	676 (29.8)	696 (23.1)	
Depressive symptoms		2.16 (0.047)	2.72 (0.072)	2.16 (0.053)	25.60 (<0.001)

**Table 3:** Factors influencing depressive symptoms by job type by complex sample linear regression analysis (N = 9375).

Characteristics (Reference)		Office Work		Service Work		Labor	
		β	*p*	β	*p*	β	*p*
Gender (female)	Male	0.226	0.596	−1.487	<0.001	−2.388	<0.001
Age (60–69)	40–49	0.355	0.186	0.159	0.714	0.458	0.080
	50–59	0.084	0.698	0.361	0.309	0.035	0.837
Household income (lower)	Upper	0.451	0.136	0.153	0.656	0.408	0.071
	Middle	0.135	0.487	−0.027	0.921	0.235	0.211
Education level (≥college)	≤Middle school	0.028	0.929	−0.047	0.905	0.062	0.788
	High school	0.222	0.251	0.553	0.082	−0.064	0.694
Type of living (others)	Living with spouse	−0.152	0.791	−0.038	0.938	−0.952	0.008
Number of household members (≥3)	1	0.688	0.427	0.784	0.268	−0.058	0.877
	2	0.527	0.022	0.450	0.116	−0.102	0.497
Housing type (others)	House	−0.754	0.043	0.415	0.199	0.245	0.156
	Apartment	−0.405	0.386	0.175	0.576	0.152	0.405
Homeownership (yes)	No	−0.162	0.474	0.091	0.774	0.064	0.683
Food intake		0.001	0.508	0.001	0.047	0.001	0.996
Body Mass Index (≥25)	<18.5	−0.388	0.492	−0.357	0.698	1.551	0.004
	18.5–24.9	0.409	0.129	0.454	0.083	0.365	0.025
Weight change (no change)	Gain	−0.192	0.329	0.399	0.321	0.001	0.996
	Loss	0.187	0.626	0.629	0.086	0.413	0.025
Weight control (no effort)	Decrease	−0.037	0.887	0.228	0.513	0.077	0.629
	Maintain	0.330	0.298	−0.809	0.221	0.308	0.209
	Increase	0.071	0.742	−0.136	0.612	0.411	0.041
Drinking (yes)	No	0.217	0.399	−0.030	0.917	−0.241	0.138
Smoking (yes)	No	0.026	0.875	0.356	0.188	−0.229	0.064
Aerobic exercise (yes)	No	0.063	0.728	−0.852	0.002	−0.381	0.007
Hypertension (yes)	No	0.341	0.131	0.360	0.312	0.072	0.671
Diabetes (yes)	No	−0.785	0.020	0.239	0.645	−0.503	0.043
Dyslipidemia (yes)	No	−0.009	0.962	−0.687	0.109	0.127	0.462
Sitting time (hours/day)		0.024	0.018	0.030	0.031	0.024	0.228
Health-related quality of life		−11.965	<0.001	−15.342	<0.001	−9.684	<0.001
Subjective health (unhealthy)	Healthy	−1.691	<0.001	−1.367	0.007	−1.70	<0.001
	Moderate	−1.314	<0.001	−0.920	0.071	−1.235	<0.001
Subjective body image (obese)	Thin	−1.691	0.253	−0.540	0.237	−0.211	0.373
	Moderate	−1.314	0.575	−0.514	0.115	−0.232	0.163
Stress (feel much)	Feel less	−1.457	<0.001	−3.008	<0.001	−1.721	<0.001
R²/F/*p*		0.232/22.54/0.001		0.496/48.08/<0.001		0.358/114.94/<0.001	

## Data Availability

The KNHANES data is publicly accessible. Data used in this study can be accessed and downloaded from the KNHANES homepage (URL: https://knhanes.kdca.go.kr/knhanes/eng/index.do, accessed on 1 September 2022).
